# Comparative efficacy and safety of drug-coated balloons versus drug-eluting stents in small vessel coronary artery disease: an updated systematic review and meta-analysis of randomized controlled trials

**DOI:** 10.1186/s43044-025-00621-7

**Published:** 2025-02-26

**Authors:** Monisha Augustine, Mustafa Arain, Muhammad Saqlain Mustafa, Iman Moradi, Matthew Fredericks, Aaliya Rahman, Muhammad Afnan Ashraf, Glawish Sualeh, Rubab khan, Aqsa Saif, Haifa Arain, Dilip Baldevsingh Rajpurohit, Abdalkareem Nael Jameel Maslamani, Behrooz Shojai Rahnama, Javed Iqbal

**Affiliations:** 1 Kasturba Medical College, Manipal, Karnataka India; 2 Dow University of Health Sciences, Karachi, Pakistan; 3 Jinnah Sindh Medical University, Karachi, 75510 Sindh Pakistan; 4 Saint George’s University, True Blue, West Indies Grenada; 5 Dr. D Y Patil Medical College Hospital and Research Centre, Pune, India; 6 Sharif Medical & Dental College, Lahore, Pakistan; 7 CMH Lahore Medical and Dental College, Lahore, Pakistan; 8 Gujranwala Medical College, Gujranwala, Pakistan; 9 Davao Medical School Foundation, Davao, Philippines; 10 Cairo University, Giza, Egypt; 11 King Edward Medical University, Lahore, Pakistan

**Keywords:** Small vessel coronary artery disease, Drug-coated balloons, Drug-eluting stents, Percutaneous coronary intervention, Meta-analysis

## Abstract

**Background:**

Small vessel coronary artery disease presents challenges in percutaneous coronary intervention due to higher restenosis rates with traditional treatments. Drug-coated balloons (DCBs) offer a potential alternative, but their efficacy compared to drug-eluting stents (DES) remains debated. This meta-analysis aims to provide updated insights into the comparative outcomes of DCBs versus DES in small coronary artery disease.

**Main text:**

Following PRISMA guidelines, a systematic review identified seven randomized controlled trials (RCTs) comparing DCBs with DES for small vessel CAD. Data were extracted and pooled for analysis, assessing outcomes including target lesion revascularization (TLR), target vessel revascularization (TVR), mortality, myocardial infarction (MI), stent/vessel thrombosis, and major adverse cardiovascular events (MACE). Statistical analysis was performed using RevMan version 5.4, employing random-effects models and forest plots with odds ratios (OR) and 95% confidence intervals (CI). Among 1,808 patients across seven RCTs, no significant difference was found in TVR between DCB and DES over 3 years (OR = 0.95, 95% CI [0.58, 1.54], *p* = 0.82). While initial analyses favoured higher TLR incidence in DES, the trend shifted towards DCB over time, with a non-significant association favouring DCB at 3 years (OR = 0.51, 95% CI [0.26, 1.00], *p* = 0.05). DCB use was associated with significantly higher rates of MACE and MI at the 3-year mark (MACE: OR = 0.55, 95% CI [0.38, 0.79], *p* = 0.001; MI: OR = 0.35, 95% CI [0.17, 0.7], *p* = 0.003), while mortality rates converged between the two interventions over time. Vessel thrombosis rates were similar between DCB and DES.

**Conclusions:**

While DCBs may offer comparable efficacy to DES in terms of TVR and TLR over shorter durations, there is a concerning trend towards higher rates of MACE and MI associated with DCB use at the 3-year mark. Further research with larger sample sizes, longer follow-up durations, and consistent inclusion criteria is needed to elucidate the optimal treatment strategy for small vessel CAD. Until then, DES may be considered a safer option for managing small vessel CAD.

## Background

The prevalence of small vessel coronary artery disease among patients undergoing percutaneous coronary intervention (PCI) is significant, affecting a substantial proportion of patients, with reported rates ranging from 30 to 67% [1, 2]. Small vessel coronary arteries pose a unique challenge in interventional cardiology due to their higher propensity for restenosis than larger vessels following PCI [3]. Consequently, the management of coronary artery disease (CAD) in these vessels requires a nuanced approach, as traditional treatment modalities, such as second-generation drug-eluting stents (DES), demonstrate limited efficacy in this context [4]. The pathology of small vessel disease is characterized by atherosclerotic plaque formation within narrow coronary arteries. This intricate pathology presents unique challenges in revascularization, with higher technical failure rates observed in coronary artery bypass grafting and an increased risk of adverse events and restenosis in PCI [5].

Amidst these challenges, alternative therapeutic strategies have garnered attention in the cardiology community. Drug-coated balloons (DCBs) have emerged as a promising option. These balloons deliver lipophilic medications to the arterial wall upon inflation, providing targeted drug delivery without the need for a polymer, potentially reducing the risk of restenosis [6]. However, the comparative effectiveness of DCBs versus DES in small vessel disease remains a subject of debate. Recent evidence suggests that DCBs may be associated with a higher risk of restenosis compared to newer-generation DES [7]. Consequently, there is a compelling need for comprehensive evaluation through meta-analysis to elucidate the comparative outcomes of these interventions.

This meta-analysis serves as an update to previous research, notably the meta-analysis conducted by Sanchez et al. [8]. By systematically reviewing and synthesizing recent randomized trials, this study aimed to provide updated insights into the efficacy and safety of DCBs versus DES in the management of small coronary artery disease. By building upon existing knowledge, this study endeavours to inform clinical decision-making and optimize treatment strategies for patients with small vessel coronary artery disease.

## Methods

### Data sources and search strategy

A meta-analysis of randomized controlled trials (RCT) was conducted following the Preferred Reporting Items for Systematic Reviews and Meta-Analyses (PRISMA) 2020 guidelines [9]. A meticulous study selection process was employed to ensure the inclusion of relevant and high-quality studies. A systematic search strategy was devised and implemented across various electronic databases, including PubMed, Scopus, ScienceDirect, and Web of Science. This search was executed independently by two reviewers and encompassed controlled vocabulary using Medical Subject Headings (MeSH) terms and free-text terms pertinent to the study topic. Example search terms and concepts included "coronary artery disease," "Drug-eluting stent," "Small vessel disease," and "target lesion revascularization." The search encompassed the literature published up to March 2024 with no language restrictions applied.

### Study selection

Two independent reviewers initially screened the titles and abstracts retrieved from the literature search. Articles deemed potentially relevant or with unclear eligibility were subjected to a full-text assessment. Any disagreements between the reviewers were resolved through discussion, with input from a third reviewer when necessary. Full-text articles were then independently reviewed based on the predetermined inclusion and exclusion criteria. RCTs were included if they compared drug-coated balloons with drug-eluting stents for the treatment of small coronary artery disease, meeting the following specific criteria: (I) availability of clinical outcome data with a follow-up duration of at least 6 months and (II) coronary artery disease with vessel diameters less than 3 mm. Studies were excluded if they were not original research articles, were conducted in non-human subjects, did not report relevant outcomes, or were followed up for less than 6 months.

### Data extraction and outcome measures

Data extraction was conducted using a standardized form to capture pertinent information from the included studies, including study characteristics, participant demographics, intervention details, and country of origin. The outcomes of interest included target lesion revascularization (TLR), target vessel revascularization (TVR), all-cause mortality, myocardial infarction (MI), stent/vessel thrombosis, and major adverse cardiovascular events (MACE). Data were pooled according to follow-up times at 6–12 months, 2 years, and 3 years.

### Quality assessment

The quality of each study’s methodology was evaluated independently by two reviewers using the Cochrane risk of bias tool for randomized controlled trials. The key domains of risk of bias that were assessed included random sequence generation, allocation concealment, blinding of participants and personnel, blinding of outcome assessment, incomplete outcome data, selective reporting, and other sources of bias. In the event of any discrepancies in the quality assessment, a third reviewer was consulted to reach a consensus. The studies were then categorized as high-, moderate-, or low-quality based on the overall assessment of their methodological quality, with a greater emphasis given to studies with high methodological quality and a low risk of bias in the synthesis of evidence.

### Statistical analysis

Statistical analysis was conducted using Review Manager (RevMan) version 5.4 (The Cochrane Collaboration, 2020). Robust methodologies were used to compare and analyse outcomes between the DCB and DES groups. Utilizing a random-effects model allowed for the calculation of pooled effects, acknowledging potential variations across studies. Forest plots featuring odds ratios (OR) and 95% confidence intervals (CIs) were meticulously crafted to visually represent major and secondary outcomes, facilitating clear interpretation of the findings. Moreover, the I2 statistic was used to assess heterogeneity, with values exceeding 50% indicating substantial variability among the studies. The significance of heterogeneity was carefully evaluated by considering the specific characteristics and nuances of each study included in the analysis.

## Results

### Literature review and study selection

**Fig. 1:**
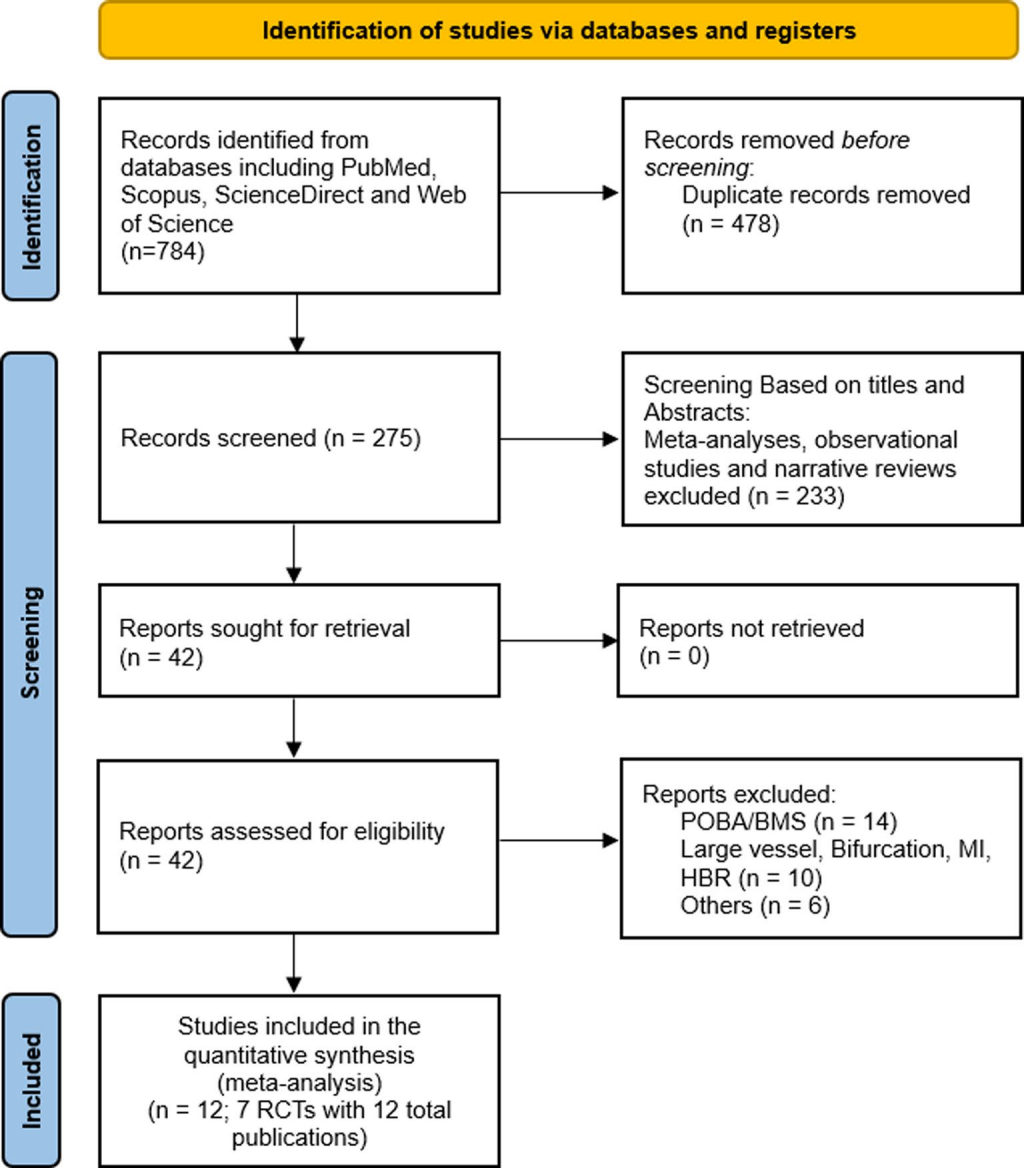
PRISMA flowchart summarizing the systematic review process according to PRISMA guidelines, showing the number of records identified, screened, assessed for eligibility, and included in the systematic review and meta-analysis

A summary of the study identification and selection through preferred reporting items and systematic analyses (PRISMA) is shown in Fig. 1. Various databases and registers were searched, yielding 784 records, of which 478 were duplicates and were hence removed. A total of 275 articles were screened, of which 233 were secondary research and observational studies. Out of the remaining 42 articles, 7 RCTs with a total of 12 publications fulfilled the eligibility criteria (Fig. 1).

### Study details

The selected randomized controlled trials fulfilling the eligibility criteria are namely Cortese et al. 2010 (PICCOLETO) [10], Latib et al. 2012 (BELLO) [3], its 2-year follow-up Naganuma et al. 2015 [11] and 3-year follow-up Latib et al. 2015 [12], Poerner et al. 2014 [4], Jeger et al. 2018 (BASKET-SMALL 2) [13] and its 3-year follow-up article Jeger et al. 2020 [6], Tang et al. 2018 (RESTORE SVD) [14] and its 2-year follow-up article Tian et al. 2020 [15], Cortese et al. 2020 (PICCOLETO II) [16] and its 3-year follow-up article Cortese et al. 2023 [17], and, lastly, Liu et al. 2023 (DISSOLVE SVD) [18]. The study characteristics of the seven main articles are summarized in Table 1**.**

**Table 1:** Study Characteristics

Author		Cortese et al	Latib et al	Naganuma et al	Latib et al	Poerner et al	Jeger et al	Jeger et al	Tang et al	Tian et al	Cortese et al	Cortese et al	Liu et al
Year		2010	2012	2015	2015	2014	2018	2020	2018	2020	2020	2023	2023
Trial Name		PICCOLETO	BELLO			–	BASKET-SMALL 2		RESTORE SVD		PICCOLETO II		DISSOLVE SVD
Country		Ospedale della Misericordia in Grosseto, Italy	15 Italian centres			University Hospital of Jena, Germany	14 clinical sites in Germany, Switzerland, and Austria		12 Chinese sites		ASST Fatebenefratelli-Sacco, Milan, Italy and 5 European centres		10 Chinese sites
Timeline of Data Extraction		August 2007 to August 2008	–			June 2009 to February 2011	April 10, 2012 to February 1, 2017		August 2016 to June 2017		May 2015 to May 2018		April 17, 2018 to March 2, 2019
Inclusion Criteria		Age ≥18 years SA or UnSA Clinical indication for PCI of ≥ 1 small coronary artery	Age ≥18 years SA or UnSA or documented silent ischaemia ≤2 target lesions LL <25 mm RVD <2.8 mm			Clinical indication for elective PCI as per guidelines Suitable lesion for angioplasty and OCT imaging.	Indication for PCI- ACS, SA, silent ischaemia Proper predilatation without higher-grade dissections, decreased blood flow (thrombolysis in myocardial infarction score ≤2), or residual DS > 30% RVD ≥2·0 to <3·0 mm		Only 1 lesion in the target small vessel LL <26 mm % visual DS of ≥70%, or ≥50% with evidence of ischaemia before PCI Visual RD ≥2.25 and <2.75 mm in the small vessel cohort and ≥2.00 and <2.25 mm in the VSV cohort		Age ≥18 years Hospitalized for stable CAD or ACS, with an indication for PCI LL<25 mm DS ≥70% (by investigator’s judgement by visual estimation) RVD 2.00 to 2.75 mm		Age 18- 80 years Only 1 target lesion in the small vessel LL <26mm % DS ≥70%, or ≥50% with documented MI RD ≥2.25 and ≤2.75mm in the small vessel cohort and ≥2.00 and <2.25mm in VSV cohort
Exclusion Criteria		MI ≤ 48 hrs; Unstable HD; CRI: sr. Creat >2 mg/dl; Life expectancy <2 years Known HS / contra-I to ASA, HEP, CLOP or paclitaxel; Sensitivity to contrast media that cannot be pretreated;	MI ≤ 48 hrs; LVEF <30%; Previous PCI ≤ 3m; Stroke < 6 months sr Creat. ≥2.0μmol/l; Platelet count <50,000 cells/mm; Pregnancy; Elective surgery <6m after the procedure; Contra-I / suspected intolerance to paclitaxel, ASA, thienopyridines, or iodinated contrast that cannot be pretreated; >3 vessels requiring PCI; aorto-ostial lesions; restenotic lesions; bypass graft lesions; chronic total occlusions; thrombus at target lesion; bifurcation needing 2-stent technique or with side branches >2.5 mm			–	Pregnancy; Life expectancy <12m; Enrolment in another RCT for coronary intervention; Inability to give informed consent Concomitant PCI of large lesions ≥ 3 mm in same epicardial coronary artery; PCI of in-stent restenosis;		MI < 1 week of the study; LVEF <35%; Total occlusion, bifurcation, left main lesions, or >2 non-target lesions requiring treatment		Recent STEMI <72 hrs LVEF <30%; Creat. clearance <30 ml/min Life expectancy <1 year Inability to provide oral and written informed consent or unwilling to come back for angiographic FU Index lesion at left main stem, aorto-ostial lesion; chronic total occlusion; target lesion previously treated by means of any device; severe calcification, major bifurcation tortuosity, untreatable thrombus at target lesion		MI < 1 week; Severe CHF or NYHA class IV; Severe VHD; Stroke<6 months; >2 Non-target lesions or 3 vessel lesions requiring PCI; Bifurcation with side branch diameter>2.0mm; Extensive thrombus in the target vessel;
Follow-up Duration		9 months (clx) 6 months (angio.)	6 months (both)	2 years (clx)	3 years (clx)	6 months (both)	1 year (clx)	3 years (clxl)	1 year (clx) 9 months (angio.)	2 years (clx)	6 months (clx and angio.)	3 years (clx)	1 year (clx) 9 months (angio.)
Sample Size (- loss to FU, refused angio. or other causes) = Total Size	DCB	29 = 28 (−1) at 6m angio and at 9m clinical FU	90 = 90 at 6m clinical FU = 78 (−12) at 6m angio	90 at 6m =89 (−1) at 1-year FU =88 (−1) at 2-year FU	85(−3) =83 (−2) at 3-year FU	51 out of 90 (9 pts did both DCB and DES) = 41 (−10) at 6m out of total 78	382 = 370 (−12) at 1 year	382 = 349 (−33) at 3yrs	116 = 114 (−2) at 1-year clinical FU =100 (−16) at 9m angio	116 = 115 (−1) at 2 years	118 =108 (−10) at 1 year = 105 (−13) at 6m angio	108 at 12m = 102 (−6) at 3 years	129 = 129 at 1-year clinical FU = 117 (−18) at 9m angio
	DES	31 = 29 (−2) at 6m angio and 9m clinical FU	92 = 91 (−1) at 6m clinical FU = 76 (−15) at 6m angio	91 at 6m =91 at 1-year FU =91 at 2-year FU	88 =83 (−5) at 3-year FU	48 out of 90 (9 pts did both DCB and DES) = 45 (−3) at 6m out of total 78	376 = 359 (−17) at 1 year	376 = 345 (−31) at 3 yrs	114 =114 at 1-year clinical FU = 93 (−21) at 9m angio	114 = 109 (−5) at 2 years	114 = 106 (−8) at 1 year = 104 (- 10) at 6m angio	106 at 12m = 101 (−5) at 3 years	118 =118 at 1-year clinical FU =98 (−20) at 9m angio
Device Type	DCB	Dior PCB	IN. PACT Falcon DEB (and prov. BMS)			Coroflex Blue BMS postdilated with SeQuent Please DEB	SeQuent Please		Restore DCB		Elutax SV DCB		Dissolve DCB
	DES	Taxus Libertè DES	Taxus Libertè PES			Xience V DES	Taxus element/Xience		Resolute Integrity DES		Xience EES		Resolute DES
Target Vessel Diameter (mm)	≤2.75		<2.8			2.5 - 3	2 - 3		≥2.25 and ≤2.75		2 - 2.75		≥2.25 and ≤2.75
Primary Endpoint	% DS at 6 months		In device LLL at 6 months	MACE at 2 years	MACE at 3 years	% struts without coverage	MACE		In-segment % DS at 9 months	TLF at 2 years	In lesion LLL		In-segment % DS at 9 months
DAPT Duration	ASA	Indefinitely	Indefinitely			–	ASA + CLOP / TICA / PRASU 4 wks – DCB 3m –DCB + BMS 6m – DES 6m – DCB + DES 12m – ACS		≥ 6m for all pts 12m – 93% of all pts		1m – DCB + stable CAD ≥ 6m – DES 12m – ACS		Indefinitely
	ADPr/P2Y12i	1m – PCB in SA 3m – PCB+stent 12m – UnSA or Taxus stent	CLOP (± TICLO) 1m – DEB 3m – DEB + BMS 12m – after DES										≥ 3m – DCB ≥ 6m – DES

*SA* Stable angina; *UnSA*: unstable angina; *MI* myocardial infarction; *HD* haemodynamics; *CRI* chronic renal insufficiency; *RF* renal failure; *HS* hypersensitivity; *Contra-I* contraindication; *ASA* aspirin; *HEP* heparin; *CLOP* clopidogrel; *TICA* ticagrelor; *PRASU* prasugrel; *RVD* reference vessel diameter; *DS* diameter stenosis; *LLL* late lumen loss; *TICLO* ticlopidine; *PCB* paclitaxel-coated balloon; *DEB* drug-eluting balloon; *BMS* bare metal stent; *PES* paclitaxel-eluting stent; *OCT* optical coherence tomography; *CHF* chronic heart failure; *VHD* valvular heart disease; *CAD* coronary artery disease; *ACS* acute coronary syndrome; *DAPT* dual antiplatelet therapy; *TLF* target lesion failure; *FU* follow-up; *ADPr/P2Y12i* ADP receptor/P2Y12 inhibitor or thienopyridines; *TVR* total vessel revascularization; *Clx* angiography; *Sr Creat*. serum creatinin

### Baseline characteristics

The seven studies included in this meta-analysis were published between 2010 and 2023, with five conducted in Europe and two in Asia. PICCOLETO and BELLO were conducted in Italy, Poerner et al. (2014) in Germany, and BASKET-SMALL 2 across 14 centres in Germany, Switzerland, and Austria. Additionally, PICCOLETO II was conducted in five European centres with a coordinating centre in Milan, Italy, whereas RESTORE SVD and DISSOLVE SVD were both conducted in China. Most trials conducted clinical follow-ups at 6 months, except for PICCOLETO which had a 9-month follow-up. Long-term clinical follow-up ranged from 2 to 3 years, with RESTORE SVD having a follow-up of 2 years, BASKET-SMALL 2 and PICCOLETO II having a follow-up of 3 years, and BELLO having follow-ups at both 2 and 3 years. Angiographic follow-ups were conducted at 6 to 9 months, except for BASKET-SMALL 2 which did not have angiographic follow-ups. Across all studies, the total sample size of patients receiving DCB was 915, with the largest number of cases in BASKET-SMALL 2 (382) and the smallest in PICCOLETO (29). In the DES group, there were a total of 893 controls, with the highest number in BASKET-SMALL 2 (376) and the lowest in PICCOLETO (31). Losses to follow-up were observed in both groups, with more cases lost to angiographic follow-up due to patient refusal. The average age of participants across all trials was 64.98 years. In the DCB group, the average age was 64.74 years, while in the DES group, it was 65.23 years. Notably, BASKET-SMALL 2 had the largest sample size in both groups, whereas PICCOLETO had the smallest sample size. The proportion of male participants was 74.1%, with slightly lower percentages in the DCB group (73.68%) than in the DES group (74.52%). The distribution of male participants varied across trials, with the highest percentages observed for BELLO and RESOLVE SVD. The baseline and angiographic characteristics of the included studies are shown in Table 2. A summary of outcomes comparing DCBs and DES in small vessel CAD is shown in Table 3.

**Table 2:** Baseline and Angiographic Characteristics

Study	PICCOLETO 2010		BELLO 2012		Poerner et al. 2014		BASKET-SMALL 2 2018		RESTORE SVD 2018		PICCOLETO II 2020		DISSOLVE SVD 2023	
No. of Patients	PCB *n* = 28	Taxus *n* = 29	DEB *n* = 90	PES *n* = 92	BMS + DEB *n* = 51	DES *n* = 48	DCB *n* = 382	DES *n* = 376	Restore DCB *n* = 116	Resolute DES *n* = 114	DCB *n* = 118	DES *n* = 114	Dissolve DCB *n* = 129	Resolute DES *n* = 118
Age in years	68 ± 10	67 ± 10	64.8 ± 8.5	66.4 ± 9	68.9 ± 9.5	68.2 ± 8.5	67.18 ± 10.33	68.42 ± 10.32	60.1 ± 10.5	60.5 ± 10.8	64 (48–80)	66 (50–82)	60.2 ± 9.5	60.1 ± 9.3
Male (%)	22 (78.6)	22 (75.9)	72 (80)	71 (77.2)	36 (70.6)	36 (75)	295 (77)	262 (70)	77 (66.4)	88 (77.2)	83 (70.3)	87 (76.9)	94 (72.9)	82 (69.5)
HTN (%)	21 (75.0)	20 (70.8)	72 (80)	75 (81.5)	51 (100)	48 (100)	324 (85)	332 (89)	78 (67.2)	86 (75.4)	77 (65.2)	76 (67.2)	95 (73.6)	89 (75.4)
DMT2 (%)	13 (37.9)	11 (46.4)	39 (43.3)	35 (38)	22 (43.1)	25 (52.1)	122 (32)	130 (35)	46 (39.7)	48 (42.1)	45 (38)	40 (35.4)	46 (35.7)	45 (38.1)
Dyslipidemia (%)	17 (60.7)	13 (54.2)	71 (78.9)	73 (79.3)	39 (76.5)	34 (70.8)	262 (70)	259 (70)	61 (52.6)	55 (48.2)	72 (61)	63 (55)	58 (45)	64 (54.2)
Smoker (%)	–	–	15 (16.7)	10 (10.9)	14 (27.5)	18 (37.5)	226 (61)	195 (54)	34 (29.3)	36 (31.6)	23 (19.5)	19 (16.7)	60 (46.5)	56 (47.5)
Stable Angina (%)	13 (46.4)	13 (44.8)	–	–	–	–	270 (71)	274 (73)	–	–	64 (54.2)	63 (55.7)	26 (20.2)	30 (25.4)
Unstable Angina (%)	–	–	22 (24.4)	20 (21.7)	–	–	48 (13)	42 (11)	80 (69)	81 (71.1)	17 (14.4)	18 (16)	82 (63.6)	77 (65.3)
Previous MI (%)	5 (17.9)	6 (20.7)	46 (51.1)	33 (35.9)	21 (41.2)	25 (52.1)	160 (42)	133 (35)	26 (22.4)	28 (24.6)	45 (38)	34 (30)	33 (25.6)	27 (22.9)
Previous PCI (%)	3 (10.7)	4 (13.8)	52 (57.8)	39 (42.4)	–	–	235 (62)	241 (64)	45 (38.8)	38 (33.3)	59 (50)	60 (53)	–	–
Previous CABG (%)	3 (10.7)	4 (13.8)	9 (10)	12 (13)	2 (3.9)	1 (2.1)	37 (10)	34 (9)	0 (0)	1 (0.9)	4 (3.3)	4 (3.5)	1 (0.8)	1 (0.8)
No. of Lesions	PCB *n* = 28	Taxus *n* = 29	DEB *n* = 94	PES *n* = 97	BMS + DEB *n* = 54 FU = 42	DES *n* = 51 FU = 48	DCB *n* = 382	DES *n* = 376	116	114	DCB *n* = 118 FU = 105	DES *n* = 114 FU = 104	Dissolve DCB *n* = 129	Resolute DES *n* = 119
Lesion Length ± SD (mm)	12.41 ± 5.89	11.38 ± 7.12	15.4 ± 6.2	14.4 ± 5.6	–	–	–	–	10.5 ± 4.8	10.8 ± 5.2	13.5 ± 7.3	14 ± 6.9	12.3 ± 5.5	11.5 ± 5.1
Reference Vessel Diameter ± SD (mm)	2.45 ± 0.28	2.36 ± 0.25	2.41 ± 0.34	2.41 ± 0.4	2.59 ± 0.36	2.61 ± 0.31	–	–	2.11 ± 0.27	2.21 ± 0.29	2.23 ± 0.4	2.18 ± 0.4	2.2 ± 0.26	2.21 ± 0.24
Minimal Lumen Diameter ± SD (mm)	0.48 ± 0.33	0.4 ± 0.3	0.6 ± 0.24	0.62 ± 0.22	0.69 ± 0.37	0.64 ± 0.33	–	–	0.64 ± 0.22	0.65 ± 0.26	0.82 ± 0.5	0.83 ± 0.4	0.68 ± 0.28	0.65 ± 0.26
Diameter Stenosis ± SD (% LD)	86 ± 12.1	89.14 ± 10.6	81.9 ± 9.6	83.3 ± 8.7	72.5 ± 14.4	75.3 ± 11.6	–	–	69.6 ± 9.3	71 ± 10.5	75 ± 17	76 ± 15	69.2 ± 11.4	70.6 ± 10.1
Bifurcation Lesion (%)	6 (21.4)	7 (24.1)	–	–	12 (22.2)	9 (17.6)	22 (6)	29 (8)	–	–	15 (12.7)	14 (12.3)	–	–
AHA Type B2/C Lesions (%)	17 (60.7)	20 (69)	45 (47.9)	46 (47.4)	B 39 (72.2) C 15 (27.8)	B 40 (78.4) C 11 (21.6)	–	–	44 (37.9)	46 (40.4)	–	–	–	–

**Table 3:** Summary of Outcomes Comparing DCBs and DES in small vessel CAD

Outcome	Follow-up Period	Number of events/Number of patients; (Absolute event rate %)		Odds Ratio and 95% Confidence Interval	*p* Value
		DCB	DES		
TVR	6–12 months	50/785 (6.4)	45/768 (5.9)	1.12 [0.67, 1.85]	0.67
	2 years	39/587 (6.6)	50/577 (8.6)	0.76 [0.49, 1.17]	0.21
	3 years	33/425 (7.76)	38/459 (8.28)	0.94 [0.58, 1.54]	0.82
TLR	6–12 months	36/511 (7)	25/498 (5)	1.43 [0.82, 2.52]	0.21
	2 years	12/205 (5.8)	14/201 (6.96)	0.92 [0.26, 3.29]	0.9
	3 years	15/185 (8.1)	27/184 (14.6)	0.51 [0.26, 1]	0.05
VT	6–12 months	25/803 (3.1)	23/782 (2.9)	0.9 [0.31, 2.6]	0.84
	3 years	2/484 (0.41)	10/477 (2)	0.25 [0.06, 1.03]	0.05
MI	6–12 months	11/893 (1.23)	22/874 (2.5)	0.51 [0.24, 1.07]	0.07
	2 years	18/587 (3)	28/577 (4.85)	0.63 [0.34, 1.16]	0.14
	3 years	11/484 (2.27)	30/477 (6.29)	0.35 [0.17, 0.7]	0.003
MACE	6–12 months	80/737 (10.8)	71/621 (11.4)	1.02 [0.58, 1.81]	0.93
	2 years	55/472 (11.65)	64/368 (17.39)	0.65 [0.44, 0.96]	0.03
	3 years	77/567 (13.58)	102/460 (22.1)	0.55 [0.38, 0.79]	0.001
Mortality	6–12 months	21/893 (2.3)	12/874 (1.37)	1.69 [0.82, 3.46]	0.15
	2 years	24/587 (4)	20/577 (3.46)	1.2 [0.65, 2.21]	0.56
	3 years	34/567 (5.99)	36/560 (6.4)	0.94 [0.57, 1.53]	0.79

*TVR* Target vessel revascularization; *TLR* target lesion revascularization; *VT* vessel thrombosis; *MACE* major adverse cardiovascular events; *MI* myocardial infarction; *CAD* coronary artery disease

### Quality assessment

**Fig. 2:**
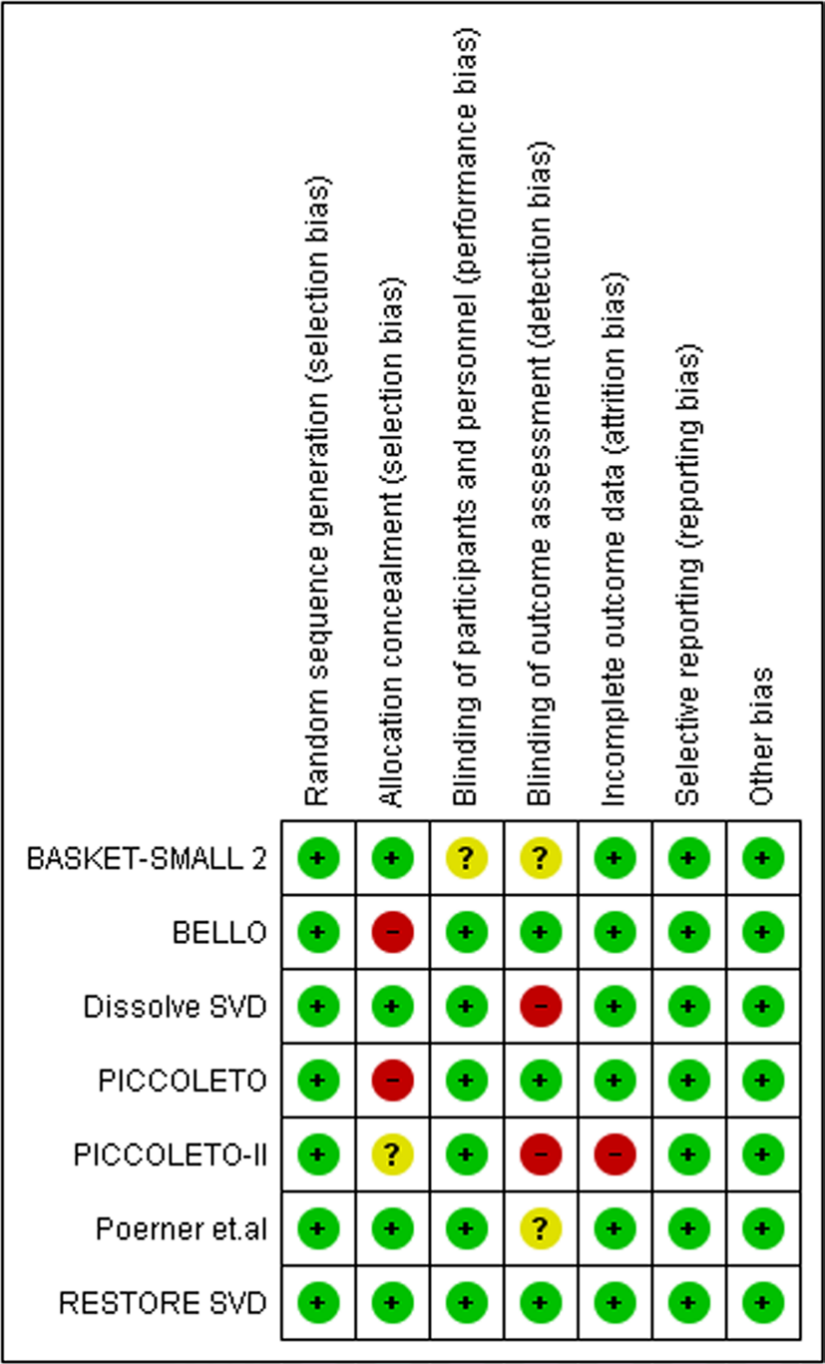
Risk of bias assessment

All seven RCTs were evaluated for the risk of bias using the Cochrane tool for Quality Assessment, as illustrated in Fig. 2. Poerner et al. and RESTORE SVD are 2 studies with the lowest risk of bias. PICOLETTO-II has the highest risk of bias, with a high risk of detection and performance bias, and an unclear selection bias. There was a 100% low risk of selection, reporting, or other bias in all seven studies. Except for BASKET-SMALL 2, which lacked effective blinding of participants or personnel, the rest of the studies had a low risk of performance bias. Detection bias had the highest unclear risk, with four studies not having specified the use of effecting blinding of outcome assessment.

### Outcomes

#### Target vessel revascularization (TVR)

**Fig. 3:**
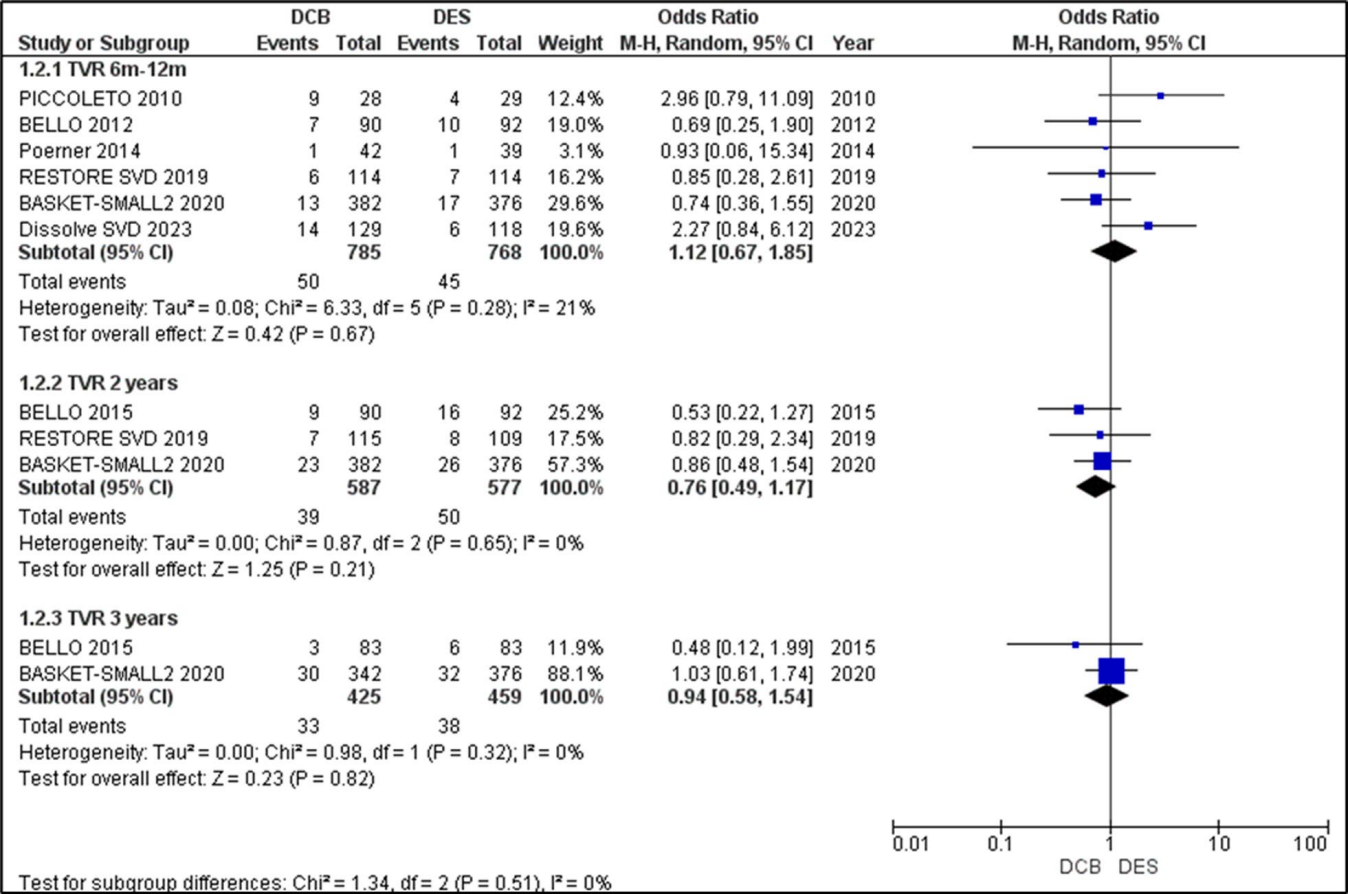
Forest plot comparing the odds ratios (ORs) for target vessel revascularization (TVR) between drug-coated balloons (DCBs) and drug-eluting stents (DES) across included randomized controlled trials

The overall TVR outcomes recorded over 3 years showed no statistical significance in either DCB or DES interventions. All studies except PICCOLETO II reported TVR outcomes for the period of 6 to 12 months. A non-significant relationship was seen for TVR in both interventions (OR = 1.12 [0.67, 1.85], p = 0.67). In the 2-year follow-up, the studies analysed were BELLO 2015, RESTORE SVD 2019, and BASKET-SMALL 2 2020. Analysis of the TVR outcomes resulted in an OR = 0.76 [0.49, 1.17], p = 0.21, with a slight favour towards DCB, though not significant. The 3-year follow-up had only BELLO 2015 and BASKET-SMALL 2 2020 reporting on the outcomes of TVR, which once again showed no significance, with OR = 0.95 [0.58, 1.54], p = 0.82. Overall, there was no heterogeneity (I2 = 0%). Conclusively, the frequency of TVR was grossly the same in both the DCB and DES groups throughout the follow-up period (Fig. 3).

#### Target lesion revascularization (TLR)

**Fig. 4:**
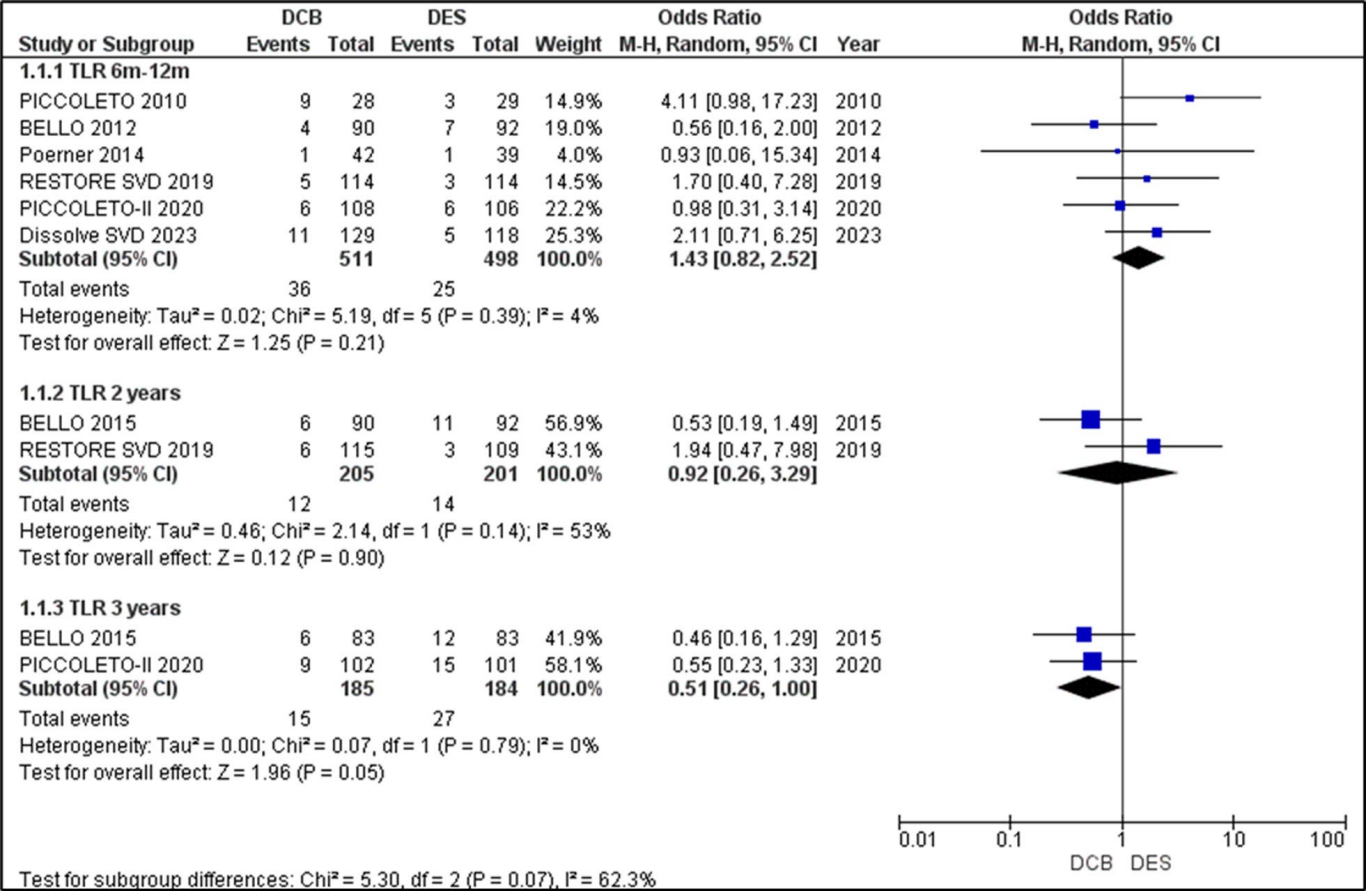
Forest plot comparing the odds ratios (ORs) for target lesion revascularization (TLR) between drug-coated balloons (DCBs) and drug-eluting stents (DES) across included randomized controlled trials

There was no statistical significance noted in the outcomes of TLR in the DES and DCB interventions for all follow-up periods up to three years. All studies, except for BASKET-SMALL 2, reported TLR outcomes in the 6–12-month period. The analysis of the TLR event rates revealed an OR = 1.43 [0.82, 2.52], *p* = 0.21, favouring a higher incidence in DES over DCB. The 2-year follow-up analysed TLR data from BELLO 2015 and RESTORE SVD 2019, presenting an OR = 0.92 [0.26, 3.29], *p* = 0.90, trending towards DCB. This shift from DES to DCB is attributed to BELLO 2015 and was also the cause of moderate heterogeneity (I2 = 53%, *p* = 0.14). In the 3-year follow-up, BELLO 2015 and PICCOLETTO II 2020 reported TLR outcomes, which when analysed showed OR = 0.51 [0.26, 1.00], p = 0.05, favouring DCB over DES, showing a non-significant relationship with no heterogeneity. Although not statistically significant, the outcome of TLR was initially higher in DES, but slowly became more prominent in DCB when followed up over 3 years (Fig. 4).

#### Vessel thrombosis (VT)

**Fig. 5:**
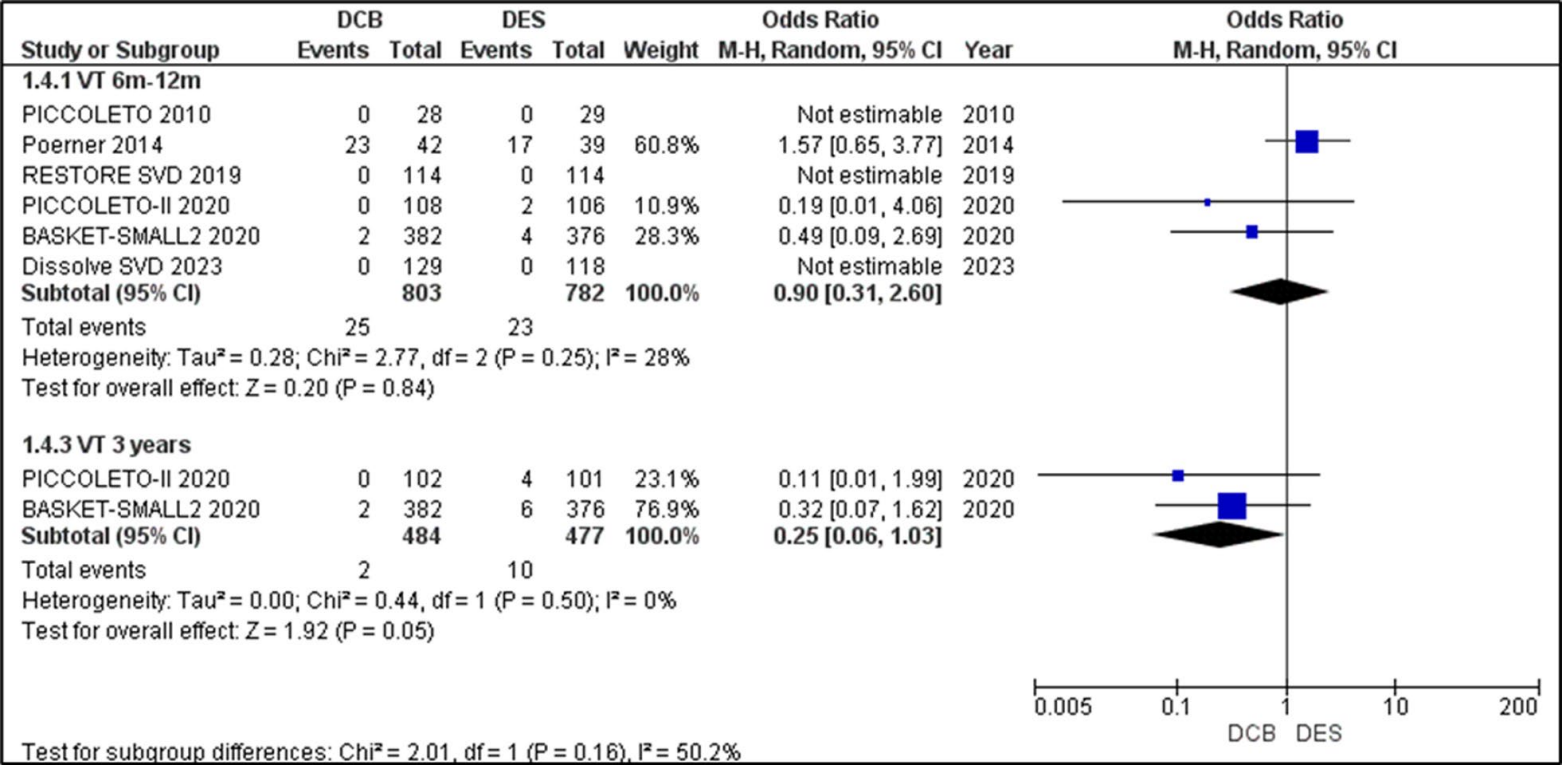
Forest plot showing the odds ratios (ORs) for vessel thrombosis (VT) risk between drug-coated balloons (DCBs) and drug-eluting stents (DES) across included randomized controlled trials

Except for BELLO, all other studies reported on the outcomes of VT in the 6–12-month periods. Among these studies, Poerner et al., PICCOLETO II, and BASKET-SMALL 2 reported a nonzero outcome for VT. The Odds ratio for VT over 6–12 months was 0.9 [0.31, 2.6], *p* = 0.84, with low heterogeneity (I2 = 28%, *p* = 0.25). This showed an equal incidence of VT in both groups, although the difference was not statistically significant. In the 3-year follow-up, PICCOLETO II and BASKET-SMALL 2 2020 reported on VT outcomes, which when analysed gave an OR = 0.25 [0.06, 1.03], *p* = 0.05, indicating a stronger association with DCB, though not significant. This follow-up period had no heterogeneity. The overall I2 = 50.2% was due to Poerner et al., which was the lone study showing high VT outcomes in DES. Conclusively, the incidence of VT was higher in the DCB group than in the DES group over 3 years (Fig. 5).

#### Myocardial infarction (MI)

**Fig. 6:**
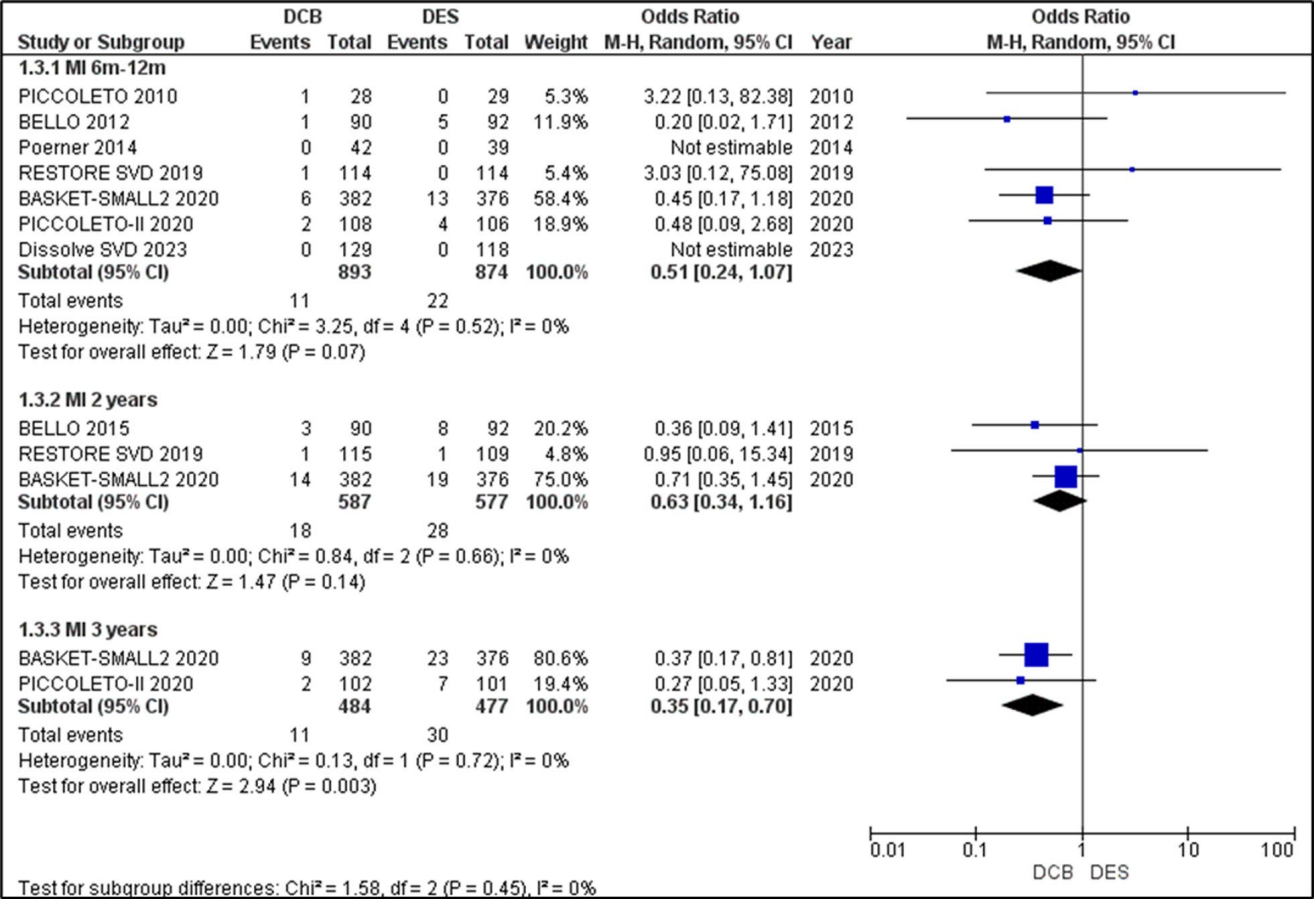
Forest plot showing the odds ratios (ORs) for myocardial infarction (MI) risk between drug-coated balloons (DCBs) and drug-eluting stents (DES) across included randomized controlled trials

All seven studies reported on the outcome of MI in the 6–12-month period. Analysis of the event rate showed OR = 0.51 [0.24, 1.07] with *p* = 0.07, indicating a higher MI incidence in the DCB group, although it was insignificant. While PICCOLETO and RESTORE SVD lean towards more MI in the DES group, the significant weightage of BASKET-SMALL 2 due to its sample size shows a stronger association of MI in the DCB group. There was no heterogeneity. The 2-year follow-up of MI events was reported in BELLO, RESTORE SVD, and BASKET-SMALL 2, the analysis of which yielded an OR = 0.63 [0.34, 1.16], *p* = 0.14, with I2 = 0. The 3-year follow-up of MI events was reported in BASKET-SMALL 2 2020 and PICCOLETO II. Analysis of the data revealed a significant association of MI events in the DCB group, with OR = 0.35 [0.17, 0.7], *p* = 0.003, with no heterogeneity. Overall, the incidence of MI over the course of 3 years was significantly higher in the DCB group than in the DES group (Fig. 6).

#### Major adverse cardiac events (MACE)

**Fig. 7:**
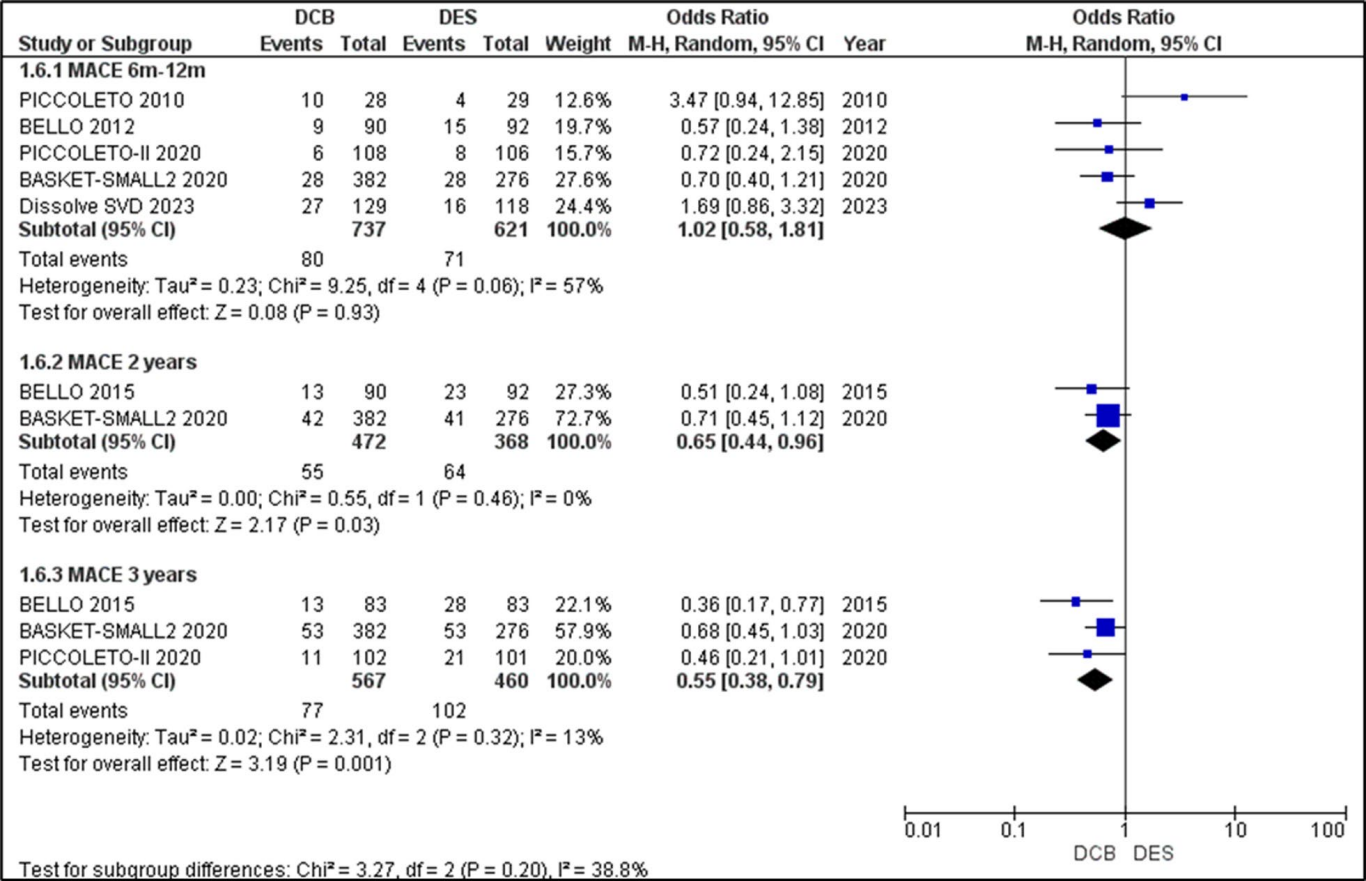
Forest plot showing the odds ratios (ORs) for major adverse cardiac events (MACE) risk between drug-coated balloons (DCBs) and drug-eluting stents (DES) across included randomized controlled trials

PICCOLETO I and II, BELLO, BASKET-SMALL 2, and DISSOLVE SVD reported outcomes of MACE in the 6–12-month period. The analysis of the MACE yielded an OR of 1.02 [0.58, 1.81], *p* = 0.93. There was moderate heterogeneity (I2 = 57%) due to PICCOLETO, which showed higher odds of MACE in DES than in DCB. In the 2-year follow-up, BELLO 2015 and BASKET-SMALL 2 2020 reported MACE, which on analysis yielded an OR = 0.65 [0.44, 0.96], with *p* = 0.03. I2 = 0%. This showed a higher incidence of MACE in DCB, with statistical significance. The 3-year follow-up of MACE was reported by BELLO 2015, BASKET-SMALL 2 2020, and PICCOLETO II, which on analysis yielded an OR = 0.55 [0.38, 0.79], *p* = 0.001, with low heterogeneity I2 = 13%. This indicates a statistically significant higher incidence of MACE in DCB than in DES. Overall, the outcome of MACE, while initially having similar odds in the first year, its association with DCB grows stronger and more significant over 3 years (Fig. 7).

#### All-cause death/mortality

**Fig. 8:**
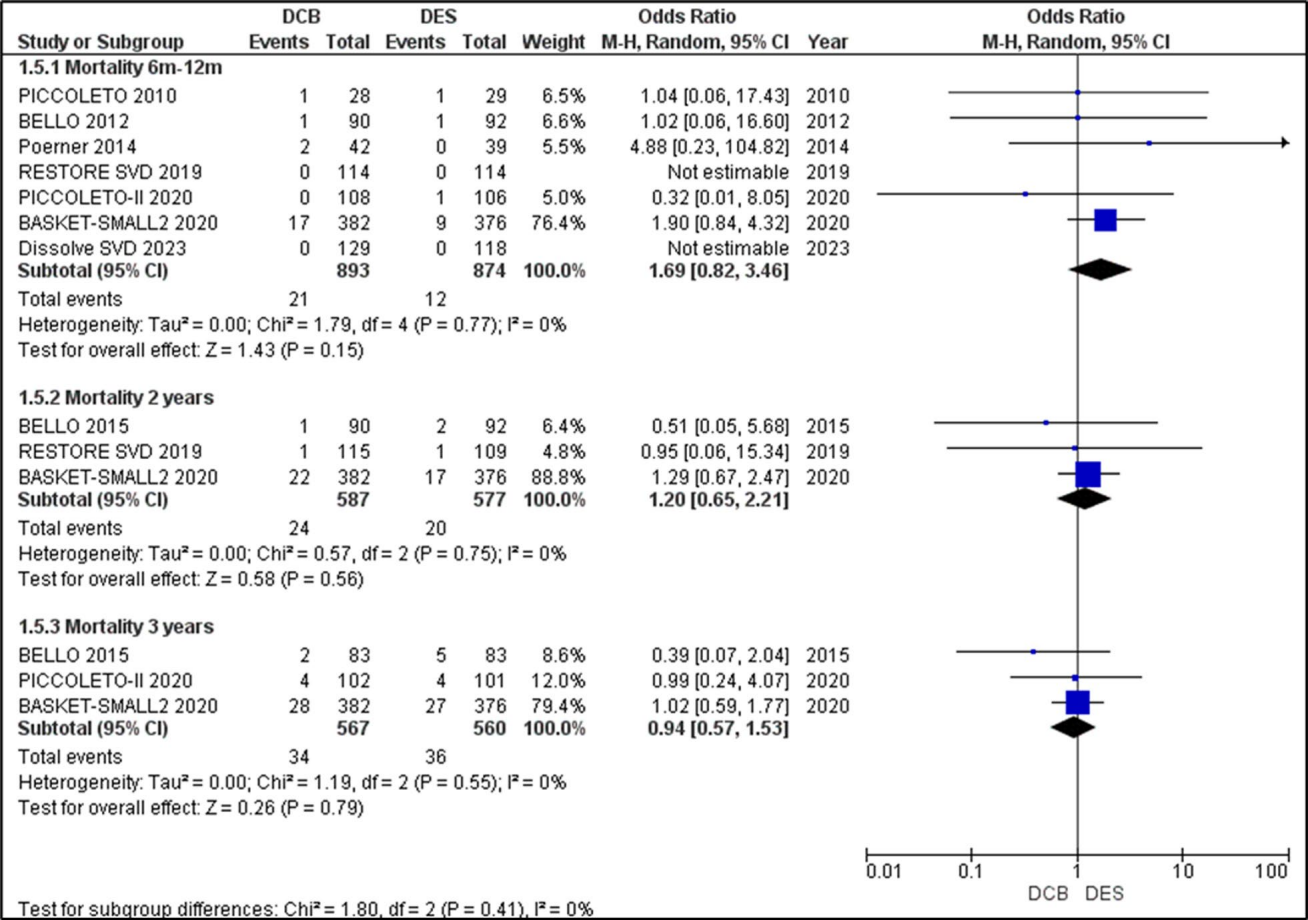
Forest plot showing the odds ratios (ORs) for all-cause mortality rates over time between patients receiving drug-coated balloons (DCBs) and drug-eluting stents (DES) across included randomized controlled trials

All seven studies reported on the outcomes of mortality for the 6–12-month period, although RESOLVE SVD and DISSOLVE SVD had zero mortality outcomes. The analysis of the event rates resulted in an OR = 1.69 [0.82, 3.46], *p* = 0.15, having no heterogeneity, I2 = 0, which indicates a higher incidence of Mortality in DES, though not significant. The 2-year follow-up outcomes of mortality were reported by BELLO 2015, BASKET-SMALL 2 2020, and RESTORE SVD 2019. Analysis yielded an OR of 1.2 [0.65, 2.21], *p* = 0.56, with no heterogeneity, I2 = 0%. The 3-year follow-up was reported by BELLO 2015, PICCOLETO II, and BASKET-SMALL 2 2020, which when analysed presented an OR = 0.94 [0.57, 1.53], *p* = 0.79, with no heterogeneity, I2 = 0. Overall, although not statistically significant, there is an initial higher incidence of mortality in DES that over the course of 3 years becomes equally common in both groups (Fig. 8).

## Discussion

Multiple studies have sought to determine the optimum treatment for SVD, but it remains unclear. DES is the standard treatment option, but its use results in prolonged treatment with dual antiplatelet therapy, as well as a risk of restenosis [12]. DCB is a novel treatment based on the quick delivery of lipophilic drugs (usually the highly lipophilic paclitaxel) via a single balloon inflation to the vessel wall. This technique ensures that antiproliferative drugs are employed directly into the vessel without permanent deployment of metal [19]. In a previous meta-analysis by Sanchez (2021) [8], the safety and efficacy of DCB were compared with those of DES for the treatment of small vessel CAD. However, a limitation of this analysis was the pooling of data from studies with heterogeneous follow-up durations into a single outcome measure. Additionally, the sample size was relatively modest, encompassing only five trials and 1459 patients.

This updated meta-analysis investigated the comparative long-term outcomes of novel DCB and DES for small vessel CAD intervention. We pooled data from seven RCTs encompassing 1,808 patients spanning a 13-year period. To explore potential time-dependent effects, we employed subgroup analysis based on follow-up duration.

Our findings revealed a concerning trend: Compared to DES, DCB use was associated with a statistically significant increase in the risk of MACE and MI at the 3-year follow-up. Additionally, the odds ratio (OR) for MACE at the 2-year follow-up also pointed towards a potential risk difference. However, no significant difference was observed between DCB and DES in terms of MI and MACE at shorter follow-up intervals.

While our findings regarding MI align with Sanchez et al. [8], this meta-analysis offers a more comprehensive analysis by incorporating MACE as an additional outcome measure. Furthermore, our investigation possessed greater statistical power due to the larger pool of RCTs (*n* = 7) and longer follow-up duration compared to previous studies.

Our analysis did not detect a statistically significant difference in the primary efficacy outcome, TVR, between the novel DCB and DES. This aligns with the findings reported by Megaly et al. [20], who observed similar rates of TVR for both interventions at the 1-year follow-up. While our initial analysis indicated a trend towards a slightly stronger association with DES for TVR at 1 year, this trend appeared to shift towards DCB at the 2-year mark. Notably, by the end of the 3-year follow-up period, the TVR rates between the two intervention groups converged, leading us to conclude that both DCB and DES resulted in comparable TVR rates over longer durations.

Our results align with those of Cai et al. [21], which indicate similar TLR rates for novel DCB and DES in treating SVD. Notably, our analysis extends this observation by demonstrating this similarity up to the 3-year follow-up mark, whereas Cai et al. [21] reported similar findings only at shorter durations. Interestingly, both our study and Cai et al. [21] identified a non-significant trend towards a higher TLR rate in the DCB group. This intriguing trend suggests potential mechanistic differences between DCB and DES. One possibility is the delayed antiproliferative effect of the drugs used in DCB compared to DES. Additionally, the lack of a permanent scaffold in DCB, unlike DES, might contribute to later vessel remodelling and potentially lead to increased TLR at longer follow-up intervals.

We observed a non-significant trend mirroring TLR findings in the outcome of mortality. Mortality appeared to be initially higher with DES than with DCB, but this difference converged by the 3-year follow-up mark. This aligns with the findings of a previous meta-analysis by Abdelaziz et al. [22], who reported similar trends in all-cause mortality between DCB and DES in the management of acute MI. These observations suggest that neither DCB nor DES offers a clear advantage in terms of mortality risk at short or long follow-up durations in our SVD patient population.

Several prior studies [23–25] have established a link between small vessel disease and an increased risk of VT following DES implantation. Our findings, however, demonstrated a similar VT risk for both novel DCB and DES at the 3-year follow-up mark, despite a trend towards a stronger association with DCB. This contrasts with the findings of Sanchez et al. [8], who reported a reduced VT risk with DCB. At the 1-year follow-up, our study observed a comparable VT risk between the two interventions.

Our analysis reports moderate heterogeneity for the outcomes of VT and TLR, which showed variance between the results of the studies. An explanation of these findings could be that Poerner et al. [4] and RESTORE SVD [15] used everolimus and zotarolimus, respectively, which are both second-generation DES [26], while other studies such as BELLO, PICCOLETO, and 28% of patients in BASKET-SMALL 2 used paclitaxel, a first-generation DES.

## Limitations

Our study has a few limitations. Of the two outcomes that showed statistical significance, MACE and MI, BASKET-SMALL 2 made up to half of the sample size, potentially distorting the result due to its questionable quality assessment and risk of bias. Due to the lack of individual baseline patient data, we were not able to analyse vessel lumen size before and after treatment to determine its effect. Furthermore, for 3-year follow-up of TVR, TLR, and VT, we were only able to gather data from two studies (representing less than half of the total sample size), which might be the cause of insignificant findings. The studies included in our analyses had varying “small vessel” size cut-offs as inclusion criteria, from 2.25 to 3.0 mm, which might have influenced out findings regarding VT, since vessel size is inversely correlated with risk of restenosis [27, 28]. Furthermore, our study included first-generation as well as second-generation DES, which might have skewed the findings. We recommend more studies of DCB vs. second-generation DES, with longer clinical follow-up durations, having greater sample size, as well as consistent small vessel CAD diameter of less than 2.5 mm [25] to not only increase the power of our findings, but also increase sensitivity in detecting small event rate for the outcome of mortality.

## Conclusion

In conclusion, this meta-analysis presents updated insights into the comparative efficacy and safety of DCBs versus DES for the management of small vessel CAD. Our findings suggest that while DCBs may offer comparable efficacy in terms of TVR and TLR over shorter follow-up durations, there is a concerning trend towards higher rates of MACE and MI associated with DCB use at the 3-year mark. Additionally, our analysis indicated a converging mortality risk between the two interventions over time. Despite these insights, this study highlights the need for further research with larger sample sizes, longer follow-up durations, and consistent inclusion criteria to elucidate the optimal treatment strategy for small vessel CAD. Until such studies are conducted, DES may be considered a safer option for managing small vessel CAD.

## Data Availability

Not applicable.

## Abbreviations

CAD: Coronary artery disease
PCI: Percutaneous coronary intervention
DCB: Drug-coated balloon
DES: Drug-eluting stent
TVR: Target vessel revascularization
TLR: Target lesion revascularization
MACE: Major adverse cardiac events
MI: Myocardial infarction
VT: Vessel thrombosis
RCT: Randomized controlled trial
